# Tropheryma whipplei-induced plastic bronchitis in children: a case report

**DOI:** 10.3389/fped.2023.1185519

**Published:** 2023-06-07

**Authors:** Xuefeng Jin, Caiyun Zhang, Chao Chen, Xiaoning Wang, Jing Dong, Yuanyuan He, Peng Zhang

**Affiliations:** ^1^ Department of Gastroenterology, Hangzhou Children's Hospital, Hangzhou, China; ^2^ Pediatric Intensive Care Unit, Hangzhou Children's Hospital, Hangzhou, China

**Keywords:** tropheryma whipplei, plastic bronchitis, children, piperacillin-tazobactam, neat-generation sequencing

## Abstract

This article reports a case of a 7-year-old child with severe pneumonia whose chest CT showed pulmonary consolidation, and bronchoscopy revealed plastic bronchitis. The metagenomic Next-Generation Sequencing (NGS) of the pulmonary lavage fluid suggested the infection of Tropheryma whipplei (T whipplei). The patient was treated with bronchial lavage to remove sputum plugs, intravenous azithromycin, and piperacillin-tazobactam and was discharged after eight days of hospitalization without any recurrence during follow-up. This article aims to raise clinical awareness of T whipplei infection and suggests that NGS for rare pathogens should be performed early for unexplained plastic bronchitis.

## Introduction

Tropheryma whipplei (T whipplei) is a Gram-positive bacillus related to actinomycetes, commonly found in the environment. However, it was not successfully cultured in macrophages until 1997 (1). Before that, diseases caused by T whipplei were often misdiagnosed or missed, but with the development of detection technology, the number of reported cases of T whipplei has recently increased. T whipplei infection is a chronic infectious disease that mainly affects the digestive system and rarely affects the lungs. No reports of Tropheryma whipplei-induced plastic bronchitis were found in the literature, so we reported this case. Our case manifested as an acute lung infection, and fiberoptic bronchoscopy confirmed plastic bronchitis.

## Case report

A 7-year-old boy was admitted to the hospital for a fever with a maximum temperature of 38.1°C and paroxysmal coughing, dyspnea, and cyanosis of the lips for two days, without spasmodic coughing, wheezing, digestive symptoms, or joint pain. Self-measured SPO_2_ was 89%. Therefore, he was admitted to a hospital and underwent intravenous administration of amoxicillin and clavulanate potassium for one day, meropenem once, and nebulization, mask, and high-flow oxygen therapy. Still, his condition did not improve, so he was transferred to our hospital for treatment.

Past medical history: The patient had a history of multiple episodes of wheezing and “asthma “ but had not experienced wheezing attacks in the past two years. He underwent tonsil and adenoidectomy surgery three years ago. There was no history of immunodeficiency, no use of immunosuppressive drugs, and no contact with patients infected with T whipplei.

Physical examination on admission: temperature 38.1°C, pulse 136 bpm, respiratory rate 34 bpm, blood pressure 118/67 mmHg, SPO_2_ 96% (mask oxygen inhalation 6 L/min), clear consciousness, low spirit, rapid breathing, three depressions sign, low breath sounds in the left lung, no rhonchi and crackles heard in both lungs and no abnormalities found in cardiac and abdominal examinations.

**Figure 1:**
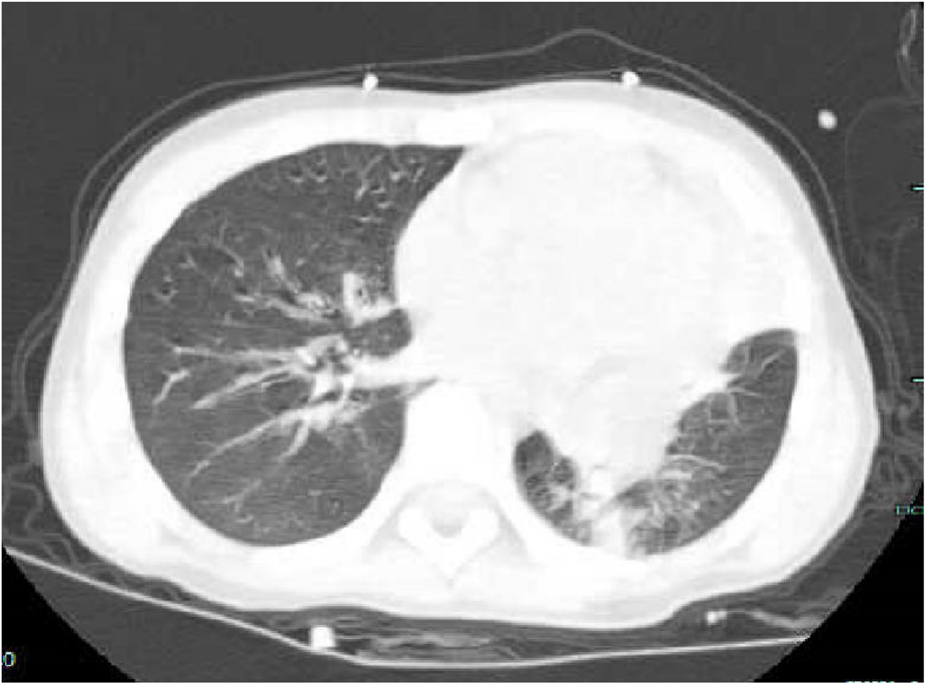
Ct scan on the day of admission (complete consolidation of the left upper lobe, bronchial obstruction of the left upper lobe, and large areas of high-density shadows in the left lower lobe).

The laboratory test in another hospital revealed blood routine: white blood cell 20.1 × 10 ^9^/L, neutrophil 92.6%, lymphocyte 4.3%, C-reactive protein 1.2 mg/L. Blood gas analysis in another hospital showed a PH 7.387, PaCO_2_ 39.2 mmHg, PaO_2_ 56 mmHg, HCO_3_^−^ 21.9 mmol/L, BE -0.31, Lac 2.1 mmol/L. The chest CT from our emergency department revealed consolidation in the left lung and occlusion of the left upper lobe bronchus (Figure 1).

After admission, several examinations were completed. Complete blood count showed white blood cell 21.11 × 10^9^/L, with 96.4% neutrophil and 2.3% lymphocyte, and C-reactive protein 11.81 mg/L. Arterial blood gas analysis showed a pH of 7.42, PaCO_2_ 34.4 mmHg, PaO_2_ 78.9 mmHg, HCO_3_^−^ 22 mmol/L, BE -1.8, and Lac 4.3 mmol/L. The patient's procalcitonin level was 0.325 ng/ml. Immunoglobulin and lymphocyte subgroup tests were normal, suggesting no immunodeficiency. Pathogen tests examinations for influenza A and B, respiratory syncytial virus, adenovirus, Chlamydia, coronavirus, rhinovirus, Mycoplasma pneumoniae, human metapneumovirus, Bocavirus, parainfluenza virus, Ureaplasma urealyticum, and Chlamydia trachomatis were negative. EB virus antibody and tuberculosis-related tests were negative. The G and GM tests were negative, and the pharyngeal swab and lavage culture was normal.

**Figure 2:**
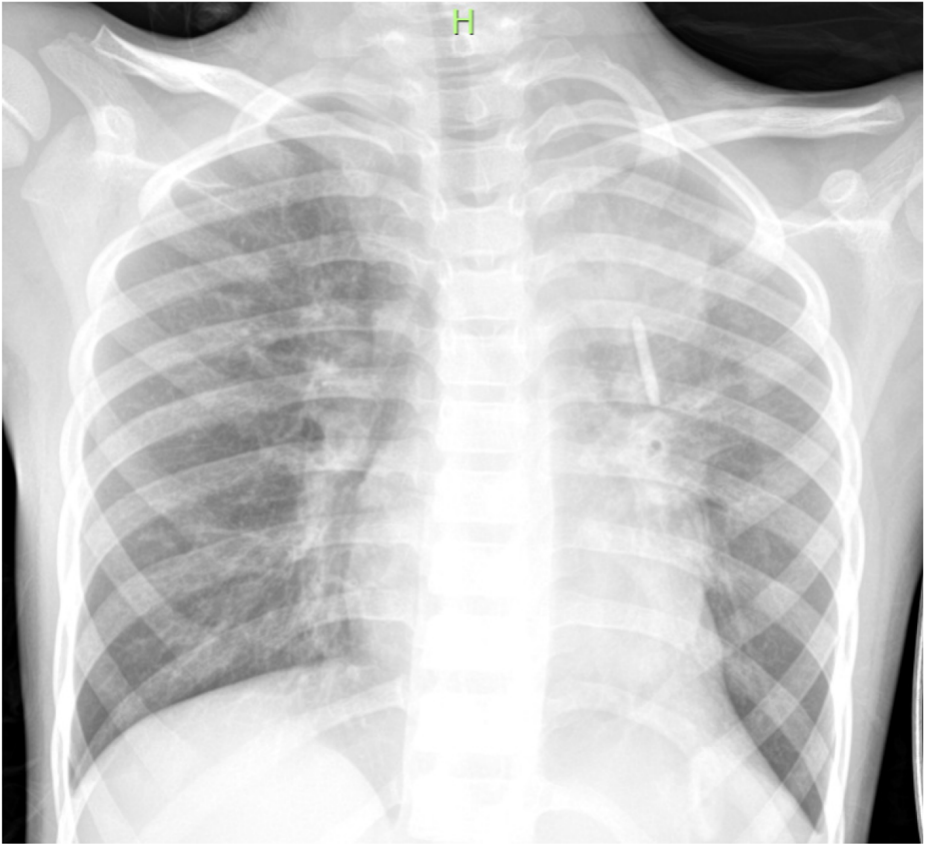
Post-bronchoscopy (increased transparency in the left lung).

**Figure 3:**
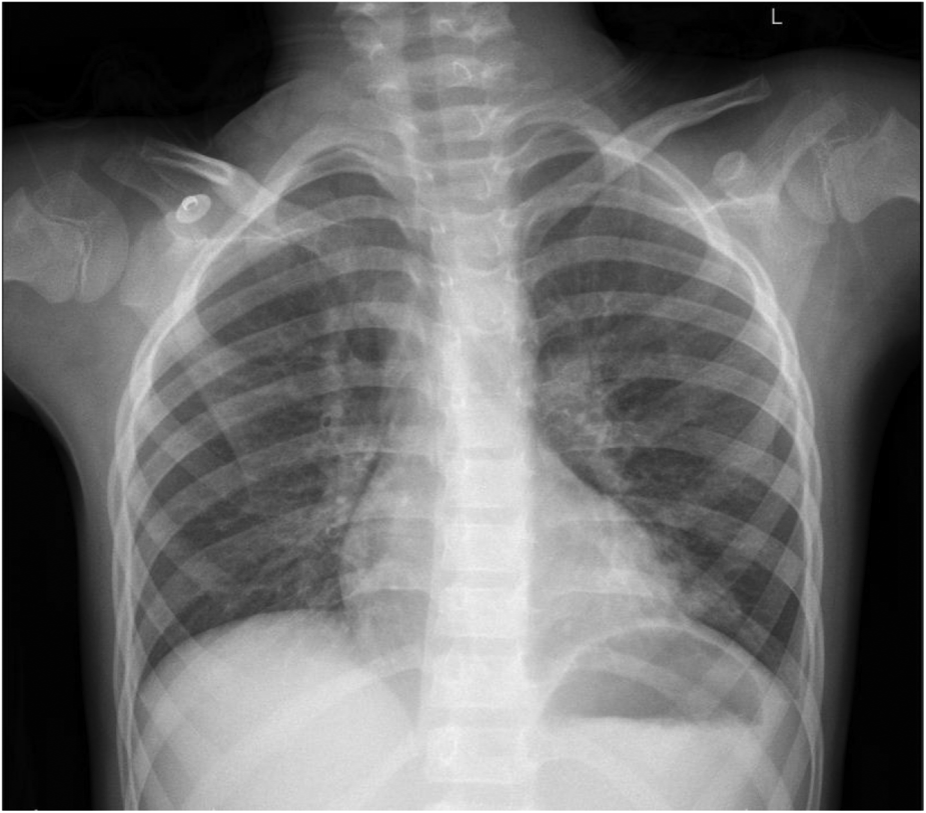
Reviewed chest x-ray before discharge (the lesion in the left lung has been mostly absorbed).

The patient received nasal high-flow warm and humidified oxygen therapy upon admission. On the same day, bronchoalveolar lavage was performed, and sputum plugs were removed. Metagenomic Next-Generation Sequencing was performed on the lavage fluid, and a follow-up chest x-ray showed increased transparency in the left lung (Figure 2). Subsequently, the patient received intravenous azithromycin to fight off infection, methylprednisolone to suppress inflammation, ambroxol to liquefy sputum, and nebulized inhalation. The results on day 3 showed T whipplei (with a sequence count of 261) and Streptococcus mitis (with a sequence count of 6) in the lavage fluid. So azithromycin was discontinued (after three days of administration) and replaced with intravenous piperacillin-tazobactam for five days (150 mg/kg.d). After five days of anti-infection treatment, the patient's blood test results were normal. The patient was discharged on the eighth day of treatment. The re-examed chest x-ray on the day of discharge suggested that the left lung lesion had significantly improved (Figure 3). The patient continued to take oral amoxicillin and clavulanate potassium for four days after discharge.

Discharge diagnoses were respiratory failure, severe pneumonia, and plastic bronchitis. One-month follow-up after discharge showed no cough and fever and normal symmetrical breathing sounds in the lungs, and the condition did not recur.

## Discussion

Plastic bronchitis is a critical condition in which a partially or completely blocked internal tube-shaped cast cause respiratory difficulties, respiratory failure, and even life-threatening situations due to impaired lung ventilation function. The gold standard for diagnosis is discovering a tube-shaped cast under bronchoscopy. In this case, the child had a fever, cough, dyspnea, and decreased SPO_2_. The diagnosis of plastic bronchitis was confirmed by bronchoscopy on the day of admission. According to literature reports, congenital heart surgery (especially after Fontan surgery), respiratory infections, bronchial asthma, and cystic fibrosis are the main causes of plastic bronchitis. Mycoplasma pneumoniae is the most common pathogen in respiratory infections (2, 3). In addition, adenovirus, influenza virus, and Bocavirus can also cause plastic bronchitis (4–6). After admission, we conducted comprehensive pathogenic examinations. However, the pathogen tests for mycoplasma pneumoniae, Chlamydia pneumoniae, influenza virus, adenovirus, bocavirus, coronavirus, respiratory syncytial virus, rhinovirus, human metapneumovirus, parainfluenza virus, tuberculosis were negative in this patient. But on the third day, the metagenomic next-generation sequencing of bronchoalveolar lavage fluid revealed T whipplei and Streptococcus mitis infection. As the number of Streptococcus mitis sequences was low, and it is generally a normal oral flora, T whipplei was the only confirmed pathogen. It should be noted that the NGS was detected one day after using antibiotics in another hospital. However, the detection targeted the pathogen's nucleic acid, and literature reports indicate it is less affected by previous antibiotics (7). Therefore, it was considered that the pathogen of pulmonary lesions, in this case, was T whipplei.T whipplei mainly infects immunocompromised individuals (8) primarily and sewage plant workers (9, 10). This patient, in this case, had no history of immunodeficiency, did not take immunosuppressants, and did not come into contact with patients infected with T whipplei. However, the patient had a history of asthma, tonsillectomy, and adenoidectomy. Further research was needed to confirm whether a history of asthma, tonsillectomy, and adenoidectomy were high-risk factors for T whipplei infection.

After T whipplei infection, the main manifestation is classic Whipple's disease, with clinical symptoms of joint symptoms, chronic diarrhea, malabsorption, weight loss, fever, skin pigmentation, lymphadenopathy, and rare involvement of the lungs, heart, and nervous system. However, there are reports of acute infections caused by T whipplei (11). In this case, the patient only had pulmonary lesions without gastrointestinal or joint symptoms, and the course of the disease was short, suggesting acute pulmonary infection caused by T whipplei. After T whipplei infection, pulmonary changes can manifest as pulmonary consolidation, patchy opacities, nodules, cavities, interstitial changes, and pleural effusion (11–13). In addition to pulmonary consolidation, bronchoscopy, in this case, revealed plastic bronchitis, which, to our knowledge, is the first case report of plastic bronchitis caused by T whipplei. The mechanism of its pathogenesis is currently unclear. Still, it may be related to the damage to the airway after T whipplei infection and subsequent immune reactions: cell and fibrinous exudate after damage to the airway epithelial cells, increased mucus exudation but decreased airway clearance, and contraction of airway smooth muscle under the stimulation of pathogens and inflammation. The combined action of various factors leads to plastic sputum plugs forming (2, 14).

Early alveolar ventilation improvement is the key to treating plastic bronchitis. We actively performed bronchoscopic lavage to remove the formed plastic sputum plugs and administered an anti-infection treatment for the Tropheryma whipplei. However, the antibacterial treatment plan for acute pulmonary infection caused by T whipplei is not yet standardized or mature (15). The reported literature suggests that penicillin, tetracycline, cefuroxime, meropenem, trimethoprim, doxycycline, and hydroxychloroquine can all be used to treat T whipplei infection (16). Before identifying the pathogen, we considered atypical pathogen infection and administered azithromycin for anti-infection treatment. After identifying T whipplei infection, we switched to administering piperacillin-tazobactam for intravenous anti-infection treatment. The patient's clinical manifestations and pulmonary signs improved significantly after treatment, and the patient was successfully discharged after eight days of hospitalization. It is worth noting that although clinical symptoms may improve after treating T whipplei infection, some cases may experience a relapse, with a reported recurrence rate of up to 20% (17). We continued to follow up with the patient for one month after discharge. We found no recurrence, indicating that bronchoscopy lavage combined with piperacillin-tazobactam is effective for treating T whipplei infection-induced plastic bronchitis in this case.

In summary, when dealing with children with plastic bronchitis and considering common pathogens, screening for T whipplei is also necessary. Second-generation sequencing can provide evidence for early pathogen identification. As for treating Tropheryma whipplei, piperacillin/tazobactam can be one of the choices for anti-infective drugs.

There are also some shortcomings in this case report. Firstly, because the patient did not exhibit symptoms such as abdominal pain or diarrhea, further diagnosis using gastrointestinal endoscopy was not conducted. Secondly, the follow-up time for this patient was short, and the subsequent recurrence situation was unclear. We will continue to track and follow up on this patient.

## Data Availability

The original contributions presented in the study are included in the article, further inquiries can be directed to the corresponding author.
